# Anthrax Lethal Factor as an Immune Target in Humans and Transgenic Mice and the Impact of HLA Polymorphism on CD4^+^ T Cell Immunity

**DOI:** 10.1371/journal.ppat.1004085

**Published:** 2014-05-01

**Authors:** Stephanie Ascough, Rebecca J. Ingram, Karen K. Chu, Catherine J. Reynolds, Julie A. Musson, Mehmet Doganay, Gökhan Metan, Yusuf Ozkul, Les Baillie, Shiranee Sriskandan, Stephen J. Moore, Theresa B. Gallagher, Hugh Dyson, E. Diane Williamson, John H. Robinson, Bernard Maillere, Rosemary J. Boyton, Daniel M. Altmann

**Affiliations:** 1 Department of Medicine, Imperial College London, London, United Kingdom; 2 Centre for Infection and Immunity, Queen's University Belfast, Belfast, United Kingdom; 3 Institute for Cellular Medicine, Newcastle University, Newcastle upon Tyne, United Kingdom; 4 Department of Infectious Disease, Erciyes University Hospital, Kayseri, Turkey; 5 Department of Medical Genetics, Erciyes University Hospital, Kayseri, Turkey; 6 School of Pharmacy and Pharmaceutical Sciences, Cardiff University, Cardiff, United Kingdom; 7 BIOMET, University of Maryland School of Medicine, Baltimore, Maryland, United States of America; 8 Defence Science Technology Laboratory, Porton Down, Salisbury, United Kingdom; 9 CEA, iBiTecS, Service d'Ingénierie Moléculaire des Protéines (SIMOPRO), Gif Sur Yvette, France; University of Illinois, United States of America

## Abstract

*Bacillus anthracis* produces a binary toxin composed of protective antigen (PA) and one of two subunits, lethal factor (LF) or edema factor (EF). Most studies have concentrated on induction of toxin-specific antibodies as the correlate of protective immunity, in contrast to which understanding of cellular immunity to these toxins and its impact on infection is limited. We characterized CD4^+^ T cell immunity to LF in a panel of humanized HLA-DR and DQ transgenic mice and in naturally exposed patients. As the variation in antigen presentation governed by HLA polymorphism has a major impact on protective immunity to specific epitopes, we examined relative binding affinities of LF peptides to purified HLA class II molecules, identifying those regions likely to be of broad applicability to human immune studies through their ability to bind multiple alleles. Transgenics differing only in their expression of human HLA class II alleles showed a marked hierarchy of immunity to LF. Immunogenicity in HLA transgenics was primarily restricted to epitopes from domains II and IV of LF and promiscuous, dominant epitopes, common to all HLA types, were identified in domain II. The relevance of this model was further demonstrated by the fact that a number of the immunodominant epitopes identified in mice were recognized by T cells from humans previously infected with cutaneous anthrax and from vaccinated individuals. The ability of the identified epitopes to confer protective immunity was demonstrated by lethal anthrax challenge of HLA transgenic mice immunized with a peptide subunit vaccine comprising the immunodominant epitopes that we identified.

## Author Summary

Anthrax is of concern with respect to human exposure in endemic regions, concerns about bioterrorism and the considerable global burden of livestock infections. The immunology of this disease remains poorly understood. Vaccination has been based on *B. anthracis* filtrates or attenuated spore-based vaccines, with more recent trials of next-generation recombinant vaccines. Approaches generally require extensive vaccination regimens and there have been concerns about immunogenicity and adverse reactions. An ongoing need remains for rationally designed, effective and safe anthrax vaccines. The importance of T cell stimulating vaccines is inceasingly recognized. An essential step is an understanding of immunodominant epitopes and their relevance across the diverse HLA immune response genes of human populations. We characterized CD4 T cell immunity to anthrax Lethal Factor (LF), using HLA transgenic mice, as well as testing candidate peptide epitopes for binding to a wide range of HLA alleles. We identified anthrax epitopes, noteworthy in that they elicit exceptionally strong immunity with promiscuous binding across multiple HLA alleles and isotypes. T cell responses in humans exposed to LF through either natural anthrax infection or vaccination were also examined. Epitopes identified as candidates were used to protect HLA transgenic mice from anthrax challenge.

## Introduction

Whether viewed as a threat to human health in anthrax endemic regions, as a bioweapon, or as a potentially devastating pathogen of livestock, there is pressing need to gain better insights into the immune response to *Bacillus anthracis*. The urgency has been underlined by recent clusters of fatal and and near-fatal anthrax infections among European intravenous drug users [1]–[4].

The *pagA*, *lef* and *cya* genes encode the three toxins associated with pathogenicity: protective antigen (PA), lethal factor (LF) and edema factor (EF). PA binds to the host cell surface receptors, tumor endothelial marker 8 (TEM8) and capillary morphogenesis gene 2 protein (CMG2) [5], [6], with recent work suggesting that α4β1- and α5β1-integrin complexes can also bind PA [7]. PA then complexes with LF to form Lethal toxin (LT), which is translocated into the host cell cytoplasm. LT is implicated in several aspects of host immune subversion. It interferes with antigen presenting cell (APC) function in the priming of adaptive immunity: expression of the co-stimulatory molecules CD40, CD80 and CD86 on dendritic cells, essential for the induction of adaptive immunity in CD4^+^ T cells, are down-regulated in the presence of LT [8]. Furthermore, LT can induce selective apoptosis of activated macrophages by disrupting the TLR dependant, p38 mediated, NF-κβ regulation and expression of pro-survival genes. LT also has a role in impairing B cell function, reducing proliferation in response to TLR2, TLR4, BCR, and CD40 [9]. Natural killer T (NKT) cells are shifted by LT from an activated to anergic state [10], [11].

Vaccination strategies in anthrax infection have been largely dominated by PA [12], [13]. For more than 40 years the major vaccines used to protect against anthrax have been the AVA (Biothrax) vaccine in the US, a filtered supernatant from the Sterne strain of *B. anthracis*, and AVP vaccine in the UK, an alum-precipitated, cell-free culture supernatant of the Sterne strain containing PA and a variable, minor, amount of LF. Both the AVA and AVP vaccines require extensive vaccination regimens, involving annual boosters. With concerns about the levels of immunity induced by these vaccines and the high rates of adverse effects [14], [15], there have been efforts to design effective next-generation vaccines with improved immunogenicity and low reactogenicity [12]. Strategies to develop recombinant protein vaccines have centered largely on PA [16]. PA based vaccines can elicit humoral immunity while avoiding the adverse reactions associated with older, filtrate based vaccines [17]–[19]. Recent vaccination programmes have investigated the impact of HLA polymorphisms, revealing considerable genetic variability in responses of human donors, notably, the very low response of HLA-DQB1*0602 individuals [20], [21].

However, the rapid decrease in humoral immune responses against PA observed in both humans and rabbits following the cessation of boosting with either filtrate based or recombinant PA (rPA) vaccines suggests that anti-PA humoral immunity induced by these vaccines may not be long-lasting [22]–[24]. The development of PA antibodies has also been shown to vary greatly within infected human populations [25], [26]. This in combination with evidence that PA-based vaccines may provide protection against lethal challenge with only select strains of *B.anthracis* [27], indicates that the induction of anti-PA antibody responses should not be the sole strategy for anthrax vaccination. Previous research has also indicated that co-immunization with a range of *B. anthracis* antigens, such as the capsular poly-γ-D-glutamic acid, surface polysaccharides, or toxins may augment the development of protective immunity [28]–[30].

Analysis of naturally-infected humans in Zimbabwe showed that most individuals mounted a response to both LF and PA [31]. We recently studied the CD4^+^ T cell immune repertoire in patients from the Kayseri region of Turkey who had become infected with *B. anthracis* and had been hospitalised for cutaneous anthrax following contact with infected livestock [32]. The study encompassed individuals who had suffered severe sepsis and undergone protracted antibiotic therapy. Contrary to expectation from our knowledge of immune subversion by LT in experimental settings, we found robust immune memory to anthrax components, with particular focus on domain IV of LF. Importantly, we were able to quantify CD4^+^ T cell memory responses in naturally exposed cutaneous anthrax patients and in AVP vaccinees, concluding that the T cell response in the former group was equally strong in response to both PA and LF, while in the latter group the major response was to LF. This prompted us to reappraise CD4^+^ T cell immunity to anthrax LF in detail.

For many microbial pathogens there is strong evidence for HLA polymorphisms as determinants of disease risk, through variable effects on the strength of immune response [33], [34]. Different HLA class II sequences vary in the anchor residues of the peptide binding groove, presenting different peptides from a given antigen, which will have an effect on the responding T cell repertoire [35]. While such studies are clearly pertinent to pathogens such as *B. anthracis* which are variably lethal to infected humans, no such analysis has been prevoiously undertaken.

In the present study, we characterize the CD4^+^ T cell immune response to LF in HLA class II transgenic mice and in infected and vaccinated humans. We observed that LF is highly immunogenic, and that specific domains and epitopes show variable immunodominance depending on HLA class II expression, with a hierarchy of response to the toxin determined by HLA class II polymorphism. This is the first time that such effects have been described in the context of anthrax. Importantly, we define highly immunodominant epitopes, common to all HLA types screened. The CD4^+^ T cell epitopes were incorporated into a peptide subunit vaccine and its protective immunity demonstrated in HLA transgenic mice following live anthrax challenge.

## Results

### Anthrax lethal factor (LF) primes strong CD4 T cell immunity with HLA-specific focus on different domains

**Figure 1:**
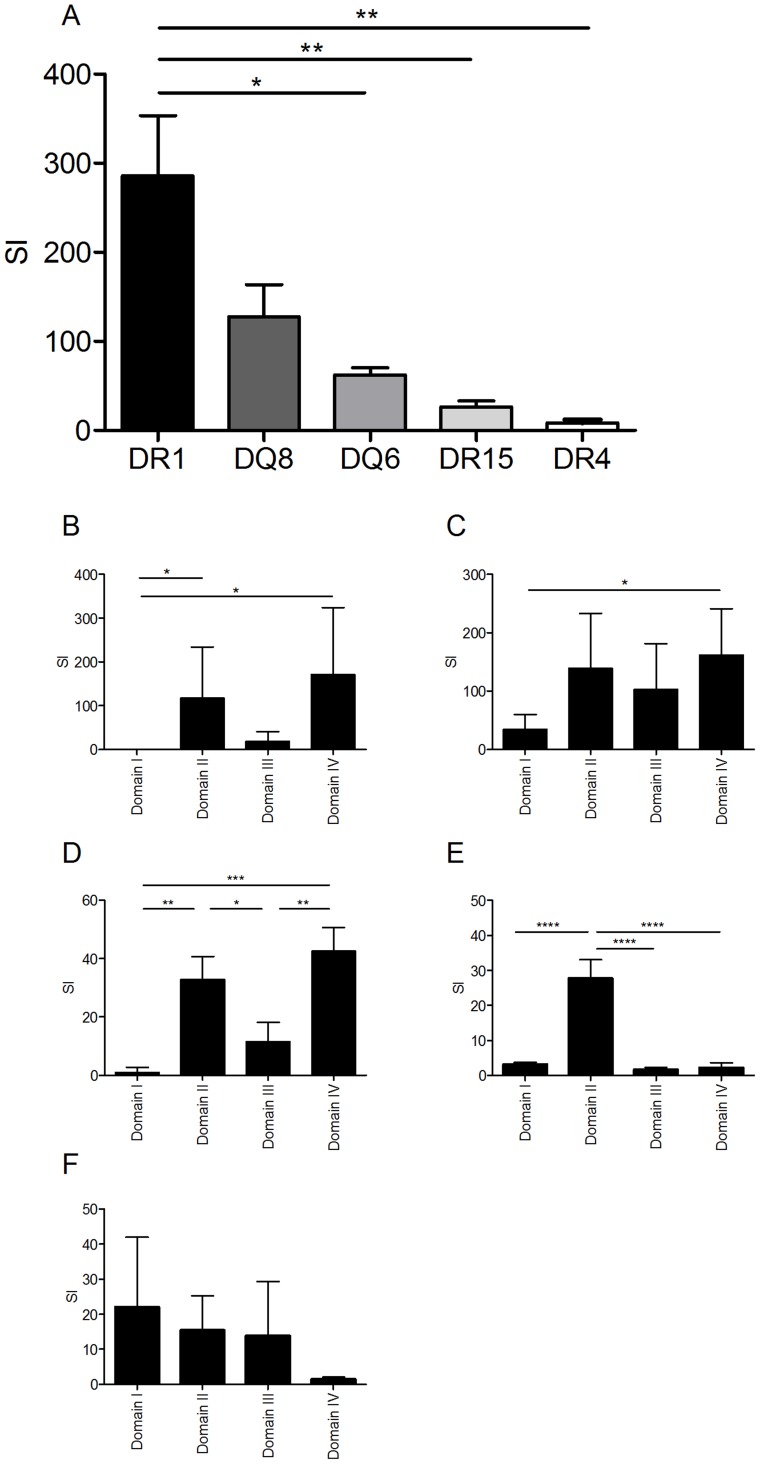
HLA transgenic mice immunized with LF generate an antigen-specific memory response to the LF protein and domains which follows an HLA hierarchy and predominantly focuses upon domains II and IV. Mice (3–5 per HLA transgenic group) were immunised in the footpad with 25 µg LF adjuvanted with Titermax Gold. Popliteal lymph nodes were harvested on day 10 and stimulated with either (A) 25 µg of whole LF or (B) the individual LF domains. The results, expressed as SI (± standard deviation) for, HLA-DR1 (n = 4), HLA-DQ8 (n = 4), HLA-DQ6 (n = 5), HLA-DR15 (n = 4) and HLA-DR4 (n = 4), demonstrated a significant difference between strains. A significantly elevated response to LF was seen in HLA-DR1 compared to DQ6, DR15 and DR4 (p = 0.0013, One-way ANOVA, with Bonferroni's multiple comparison). Cell cultures from mice transgenic for (B) DR1, (C) DQ8, (D) DQ6, (E) DR15 and (F) DR4 were stimulated for 3–5 days with LF domains; proliferation was measured by ^3^H-thymidine incorporation after 5 days stimulation (B, C & D), IFNγ production was assayed by ELISpot development and enumeration after 3 days stimulation (E & F). In HLA-DR1 transgenics (B) responses to domain I were significantly lower than responses to domains II and IV (p = 0.0081, Kruskal-wallis, with Dunn's multiple comparisons), while HLA-DQ8 transgenics (C) only showed a significant difference in response between domains I and IV (p = 0.0174, Friedman with Dunn's multiple comparisons). The response to the individual domains in HLA-DQ6 (D) showed significant variance (p = 0.0002, One-way ANOVA with Tukey's multiple comparisons) with the responses to domains II and IV significantly greater than the responses to domains I or III. The response to the individual domains in HLA-DR15 (E) also differed significantly (p<0.0001, One-way ANOVA with Tukey's multiple comparisons), however, only the response to domain II was elevated compared to domains I, III and IV). Data is represented as the stimulation index (SI) calculated as the mean cpm or IFNγ production of triplicate wells in the presence of peptide divided by the mean cpm or IFNγ production in the absence of antigen. Results are given as the mean ± SD/SEM.

Different HLA class II molecules vary in their peptide binding specificity and so present different peptides of a given antigen, with consequences for the CD4^+^ T cell repertoire activated during the immune response. As a reductionist tool for dissecting the role of individual HLA heterodimers we used mice transgenic for each of the human HLA alleles, DRB1*0101 (HLA-DR1), DRB1*1501 (HLA-DR15), DRB1*0401 (HLA-DR4), DQB1*0302 (HLA-DQ8) and DQB1*0602 (HLA-DQ6) in the absence of endogenous MHC class II expression. Following immunization with recombinant LF, all HLA transgenic mice responded to LF protein, but responses to the four domains of which the protein is composed varied (Figure 1). Using mouse strains differing only in their expression of human HLA class II alleles, we found a pronounced hierarchy of response, with HLA-DR1 transgenics mounting a considerably larger response than HLA-DQ6, DR15 or DR4 transgenics, and HLA-DR4 transgenics showing the weakest response (Figure 1A). This was not a simple reflection of strain differences in HLA transgene expression or CD4+ positive selection, as the least responsive strain, HLA-DR4, shows the highest level of HLA class II expression (data not shown). Of particular interest with respect to diversity of outcomes during infection of outbred human populations, expression of different HLA class II alleles was associated with a focus on different domains of the LF molecule. LF immunized HLA-DR1 transgenics showed an elevated response specifically to restimulation with LF domains II and IV (Figure 1B), while the HLA-DQ8 transgenics response to domain IV was significantly elevated relative to the domain I response (Figure 1C). HLA-DR15 transgenic mice showed a significantly elevated response to domain II alone (Figure 1E), while HLA-DQ6 transgenic mice demonstrated significant responses to domains II and IV (Figure 1D). HLA-DR4 transgenics respond to all four domains (Figure 1F). All of the HLA transgenics used in this study generated a memory recall to domain II of LF (Figure 1B–F). These results confirm that HLA polymorphisms play a role in the differential response to the domains of LF, and contrast with the corresponding lack of response to LF domains in sham immunized HLA transgenic mice (Supplementary Figure S1A).

### LF contains several HLA class II binding regions including a region of exceptionally high binding affinity across distinct HLA polymorphisms

A peptide library of overlapping 20-mers representing the complete anthrax LF sequence was evaluated for binding to seven common HLA-DR alleles, DRB1*0101 (DR1), DRB1*0401 (DR4), DRB1*1101 (DR11), DRB1*0701 (DR7), DRB1*1501 (DR15), DRB1*0301 (DR3) and DRB1*1301 (DR13) (Table 1). The region of the LF sequence encompassing amino acids 457–486 contains at least 2 epitopes able to bind most or all HLA-DR alleles tested with exceptionally high affinity. A further twelve peptides, LF_101–120_, LF_171–190_, LF_241–260_, LF_251–270_, LF_261–280_, LF_457–476_, LF_574–593_, LF_594–613_, LF_604–623_, LF_644–663_, LF_674–693_ and LF_694–713_, showed strong to moderate binding across all seven HLA-DR alleles.

**Table 1:** The immunodominant LF epitopes, identified in transgenic mouse strains, show relatively broad binding to common HLA-DR alleles.

LF peptide sequence	Protein Domain	HLA-restriction based on CD4 T cell response class II HLA transgenics after priming with LF	Relative binding affinity of peptide to HLA-DR molecules						
			DR1	DR3	DR4	DR7	DR11	DR13	DR15
^61^ RNKTQEEHLKEIMKHIVKIE ^80^	I	DQ8	4810	>76	>1 539	77	**2**	**8**	>2 184
^71^ EIMKHIVKIEVKGEEAVKKE ^90^	I	DQ8	243	50	567	249	**4**	19	329
^101^ SDVLEMYKAIGGKIYIVDGD ^120^	I	DQ8	**5**	>76	305	**1**	**4**	123	**2**
^141^ YGKDALLHEHYVYAKEGYEP ^160^	I	DQ8	199	>76	168	441	**9**	**6**	4000
^151^ YVYAKEGYEPVLVIQSSEDY ^170^	I	DR1, DQ8	771	>238	555	1182	14	**3**	>262
^161^ VLVIQSSEDYVENTEKALNV ^180^	I	DR1	1702	82	29	66	168	>172	958
^171^ VENTEKALNVYYEIGKILSR ^190^	I	DR1	**1**	418	81	36	**0.03**	71	**0.2**
^181^ YYEIGKILSRDILSKINQPY ^200^	I	DQ8	69	**3**	19	**4**	**0.4**	**5**	43
^221^ LLFTNQLKEHPTDFSVEFLE ^240^	I	DQ8	909	>76	**6**	51	148	>172	193
^241^ QNSNEVQEVFAKAFAYYIEP ^260^	I	DR1	**0.1**	173	137	**0.4**	**2**	327	**1**
^251^ AKAFAYYIEPQHRDVLQLYA ^270^	I	DR1	115	39	51	97	**4**	**9**	**8**
^261^ QHRDVLQLYAPEAFNYMDKF ^280^	I	DR1, DQ8	146	**3**	100	83	**5**	267	**0.3**
^271^ PEAFNYMDKFNEQEINLSLE ^290^	II/III	DQ8	2156	>76	**2**	42	**2**	>172	463
^387^ KELLNRIQVDSSNPLSEKEK ^406^	II/III	DQ8	146	**1**	**0**	36	274	1,714	**1**
^427^ DTGGLIDSPSINLDVRKQYK ^446^	II	DR15	866	**0.3**	935	15	179	**8**	130
^457^ HQSIGSTLYNKIYLYENMNI ^476^	II	DR1, DR4, DR15, DQ8, DQ6	115	**1**	55	**3**	44	**5**	**0.02**
^467^ KIYLYENMNINNLTATLGAD ^486^	II	DR1, DR4, DR15, DQ8, DQ6	**0.2**	**1**	**0.2**	**5**	**5**	17	**0.1**
^547^ LENGKLILQRNIGLEIKDVQI ^567^	IV	DR1, DR4, DR15	**0**	**4**	**7**	**1**	**3**	**9**	**0.04**
^558^ IGLEIKDVQIIKQSEKEYIRIDAKVVP ^585^	IV	DR1, DR15	**1**	**2**	919	**7**	73	24	**1**
^574^ EYIRIDAKVVPKSKIDTKIQ ^593^	IV	DR1	**3**	**1**	15	12	21	24	156
^594^ EAQLNINQEWNKALGLPKYT ^613^	IV	DR15, DQ8	104	173	43	68	100	17	**1**
^604^ NKALGLPKYTKLITFNVHNR ^623^	IV	DR1	**0**	250	10	**4**	**2**	19	**3**
^614^ KLITFNVHNRYASNIVESAY ^633^	IV	DR1, DR4	35	**0.4**	**3**	72	**7**	**0.5**	**5**
^644^ QSDLIKKVTNYLVDGNGRFV ^663^	IV	DR15	12	26	15	**4**	139	38	**0.2**
^654^ YLVDGNGRFVFTDITLPNIA ^673^	IV	DR4	123	**0.3**	**0.3**	19	45	>2 733	**2**
^674^ EQYTHQDEIYEQVHSKGLYV ^693^	IV	DR1	**1**	245	237	**0**	35	15	13
^694^ PESRSILLHGPSKGVELRND ^713^	IV	DR15	**7**	354	22	51	120	21	17
^714^ SEGFIHEFGHAVDDYAGYLL ^733^	IV	DR15	**5**	>1 000	46	131	849	>2 733	**0.04**
^724^ AVDDYAGYLLDKNQSDLVTN ^743^	IV	DR4, DR15	1,380	42	10	1,789	55	>2 733	**0.1**
^744^ SKKFIDIFKEEGSNLTSYGR ^763^	IV	DR4	**4**	474	**0.2**	302	**0.4**	1,225	**1**

The relative binding affinity of peptides to HLA-DR molecules were expressed as a relative activity (ratio of the IC_50_ of the peptide to the IC_50_ of the reference peptide which binds strongly to the individual HLA II molecule). Peptides with a high relative binding affinity of <10 are indicated in bold. Means were calculated from at least three independent experiments.

### Characterization of LF CD4^+^ T cell epitopes demonstrates the influence of HLA-DR and HLA-DQ polymorphisms and reveals promiscuous, highly immunodominant epitopes common to distinct HLA alleles

**Figure 2:**
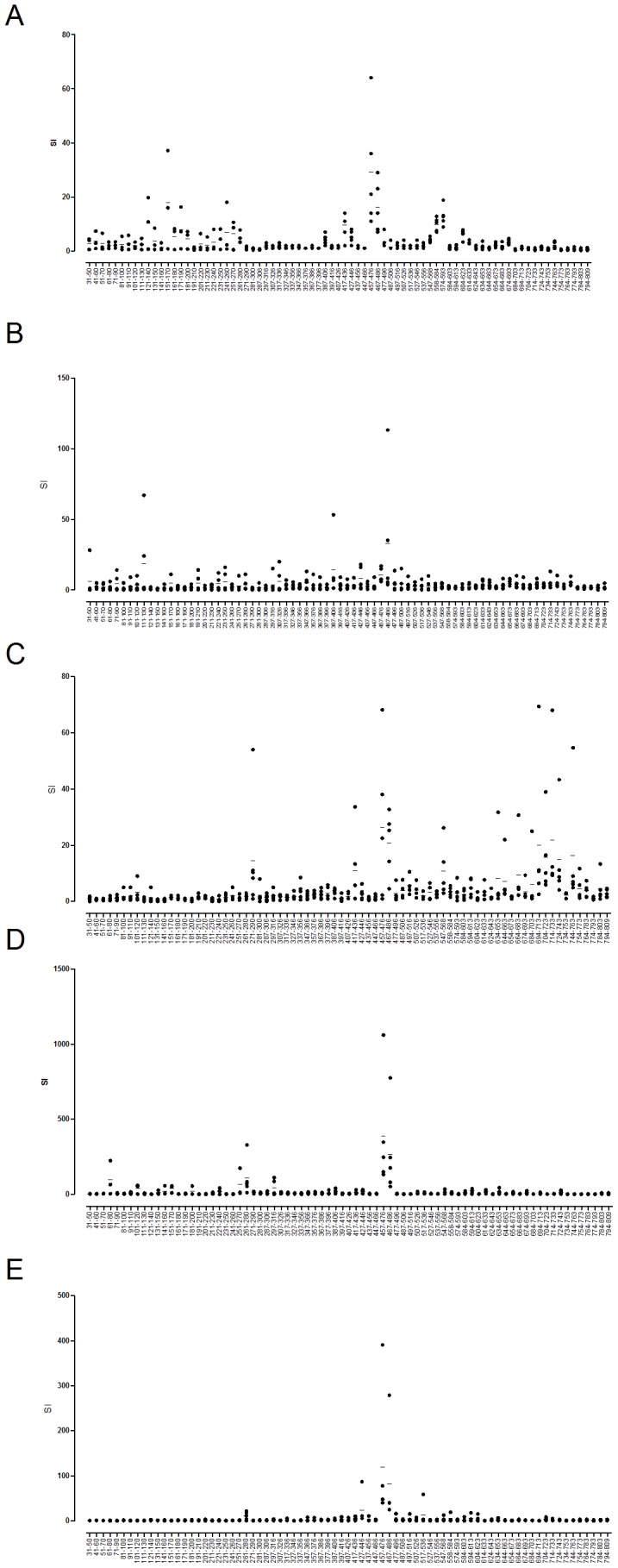
Epitope-rich, immunodominant regions of LF and epitopes common to diverse HLA polymorphisms. Popliteal lymph nodes from mice transgenic for DRB1*0101 (A), DRB1*0401 (B), DRB1*1501 (C), DQB1*0302 (D) and DQB1*0602 (E) (n = 5) were harvested 10 days after immunization with 25 µg LF adjuvanted with Titermax Gold and stimulated with 25 µg of each 10mer peptide in the LF peptide library (LF_31–809_). Responses were considered positive if the response was ≥2 SD above the cells plus medium control. Data is represented as scatter plots, showing the responses of individual mice as the stimulation index (SI) calculated as the mean cpm or IFNγ production of triplicate wells in the presence of peptide divided by the mean cpm or IFNγ production in the absence of antigen. Results are given as the mean ± SD/SEM.

The immunodominant CD4^+^ T cell epitopes within LF were mapped by immunizing HLA transgenics with recombinant LF protein and restimulating draining lymph node cells with a peptide library spanning the LF sequence. The resulting epitope maps reveal a picture of HLA-restricted epitopes in LF indicating that the immunodominant epitopes were largely localized to domains II and IV (Figure 2). The immunological memory to the LF peptides contrasted with the lack of responses to the peptides in sham immunised HLA-DR4 mice (Supplementary Figure S1B). The two epitopes shown to be exceptionally high affinity binders to diverse HLA-DR alleles, LF_457–476_ and LF_467–486_, located in domain II, not only elicited very sizeable responses, but were both recognised by all LF immunized HLA transgenics, suggesting that these epitopes were both immunodominant and promiscuous in their HLA binding. Whilst promiscuous peptides have been previously identified which bind strongly to a number of distinct HLA-DR or HLA-DQ molecules [36]–[38], the substantial differences between the binding grooves of HLA-DR and HLA-DQ isotypes [39]–[41] have resulted in the identification of a relatively low number of peptides that can be presented by such diverse isotypes [42]. These two LF epitopes, able to stimulate CD4^+^ T cells at very high frequency and across HLA class II differences are thus highly unusual and of considerable interest both for efforts to understand immunity to anthrax and to design universally stimulatory vaccines.

A number of regions that had shown strong HLA binding affinity were indeed identified as functional, immunodominant epitopes, with domain IV especially rich in epitopes able to induce a strong *in vivo* response. CD4^+^ T cell responses to the domain IV peptide, LF_547–567_, were identified in HLA-DR1, HLA-DR4 and HLA-DR15 transgenic lines, indicating that this epitope was presented solely by HLA-DR alleles. Two more domain IV epitopes, LF_724–743_ and LF_744–763_, were both HLA-DR4 and HLA-DR15 restricted. While domains II and IV contained a number of HLA-DR restricted epitopes, the majority of HLA-DQ8 restricted epitopes were found in domains I and II, and the HLA-DQ6 restricted epitopes were located only in domain II.

The greatest number of epitopes identified were DRB1*0101 restricted, with the HLA-DR1 transgenic strain recognising 14 epitopes, this was followed by 13 DQB1*0302 restricted epitopes. Ten epitopes were DRB1*1501 restricted, and 7 DRB1*0401 restricted epitopes were identified, while only 2 DQB1*0602 restricted epitopes were identified.

Some HLA-restricted peptide epitopes were identified which lay within regions of the LF protein not previously shown to elicit a response when provided as a whole protein antigen. LF immunized HLA-DR1 and HLA-DQ8 transgenics responded to peptides located within domain I, which as an intact domain did not elicit memory recall in the respective LF immunized transgenic mice (Figure 1B and 1C); similarly LF immunized HLA-DR4 and HLA-DR15 transgenics generated responses to peptide epitopes in domain IV, which also did not demonstrate a recall response following stimulation with the whole domain (Figure 1E and 1F).

**Figure 3:**
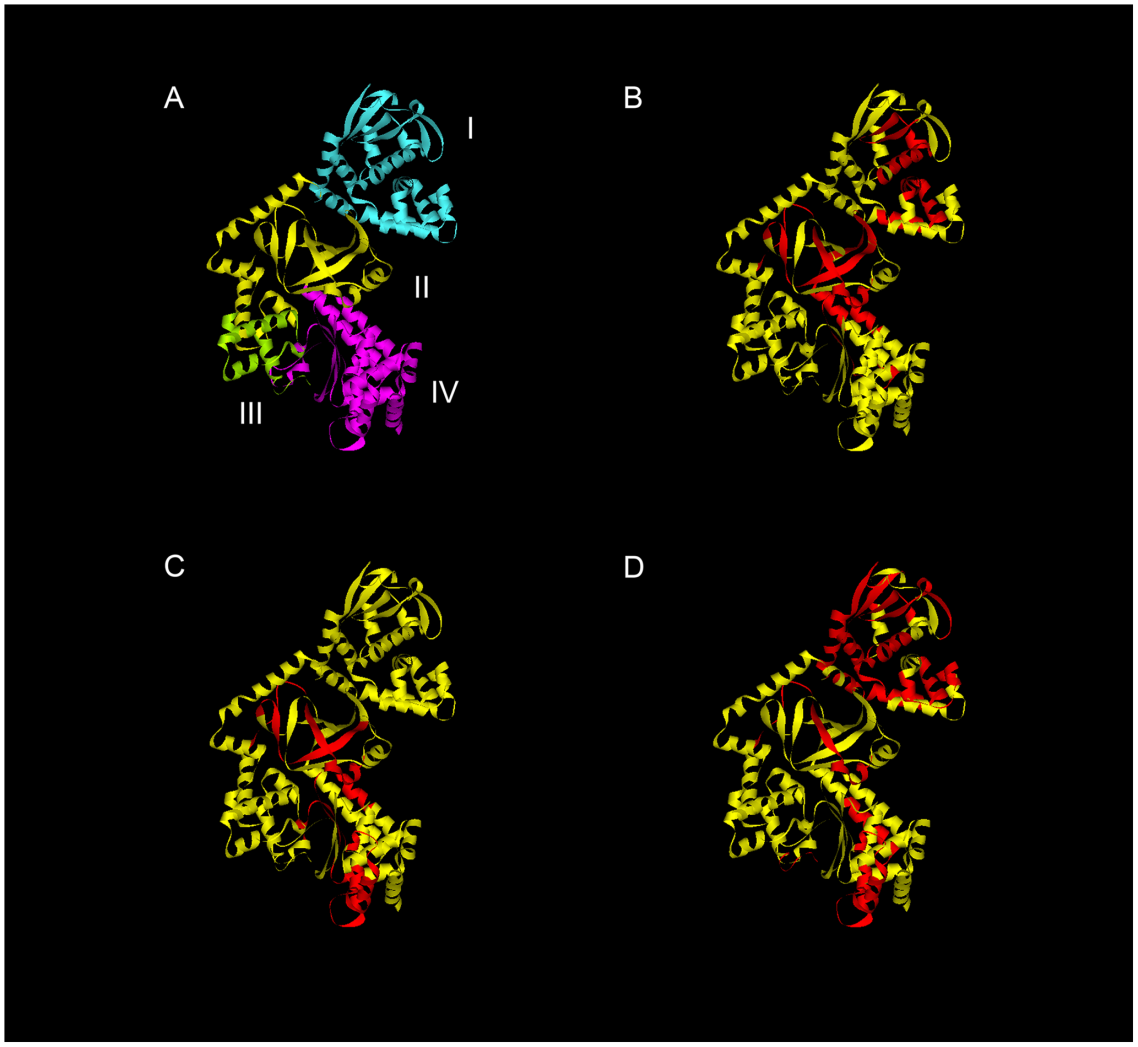
Regions HLA-DR and DQ-presented anthrax LF epitopes mapped onto the LF protein structure reveals clustering of immunogenic epitopes. The structural domains of LF protein are indicated in Roman numerals (A). Immunodominant epitopes identified in this study from mice transgenic for DRB1*0101 (B), DRB1*0401 (C), and DQB1*0302 (D) are superimposed on the LF crystal structure (Protein Data Bank accession code 1J7N). Roman numerals indicate the structural domains. Ribbon diagrams were generated using the Accelrys discovery studio client 2.5 program.

The HLA specific epitopes identified in mice transgenic for DR1, DR4 and DQB1*0302 are modeled on the LF crystal structure in Figure 3. Despite the heterogeneity which can be observed in the range of LF peptides presented by the HLA transgenics, there were identifiable areas rich in allele specific immunodominant peptides, presumably indicative of structural accessibility to cleavage by antigen processing enzymes. The T cell responses to epitopes located in the catalytically active domain IV were overwhelmingly dominated by HLA-DR presentation, as only a single DQB1*0302 restricted epitope (LF_594–613_), and no DQB1*0602 restricted epitopes, were identified in this substrate recognition and binding domain. It is also possible to identify, within the VIP2-like domain II, the cluster of epitopes containing the immunodominant peptides LF_457–476_ and LF_467–487_, which were presented by all the HLA transgenics.

Domain III, which has marked structural similarity to domain II, possibly due to its origins as a duplication of this domain, displays none of the immunogenicity associated with domain II [43], [44]. We observed no immunodominant T cell epitopes within this domain in any of the HLA transgenic strains utilised in the epitope mapping (Figures 1 and 2).

### T cell responses to LF in naturally infected anthrax patients or AVP-vaccinees

While we had previously investigated responses of human donors to epitopes within domain IV [32], it was important to obtain a comprehensive picture of the immune responses of human donors following either natural infection or vaccination. Having shown in the more reductionist context of HLA transgenics expressing single HLA class II heterodimers that LF is highly immunogenic and epitope rich, one would expect an even more complex picture in, heterozygote humans carrying multiple HLA class II isotypes. We found a heterogeneous response, spread across domains I–III of the entire protein, which was distinct according to the nature of exposure to anthrax: epitopes that were predominantly a feature of the response of vaccinees were rarely recognized by the majority of infected donors or healthy controls, and vice versa (Table 2).

**Table 2:** Frequent, large CD4 T cell epitope responses to anthrax LF domain I–III peptide panel in immune human donors.

Human cohorts	HLA class II						T cell response to anthrax LF domain I–III epitopes, SFC/10^6^ cells						
	DR1		DRB3/4/5		DQB1		LF 41–61	LF 101–120	LF 281–300	LF 337–356	LF 417–436	LF 437–456	LF 467–486
Infected donor 2	4	4	53	53	8	8	0	0	345	0	0	0	323
Infected donor 3	4	14	52	53	5	8	0	0	218	0	0	0	0
Infected donor 6	11	13	52	52	6	11	0	0	0	0	0	0	291
Infected donor 7	4	14	52	53	5	4	0	0	227	0	0	0	633
Vaccinee 3	11	13	52	52	6	7	1275	1357	0	1314	1322	1009	0
Vaccinee 4	15	7	51	53	2	6	473	509	0	0	837	451	0
Vaccinee 5	103	17	52	52	2	5	495	0	0	703	0	0	0
Vaccinee 6	1	13	52	52	5	6	0	0	0	0	0	416	0
Vaccinee 8	1	1	-	-	5	5	0	0	0	0	725	0	0
Vaccinee 10	7	15	51	53	2	6	0	423	0	521	0	0	0

Frequent, large CD4 T cell epitope responses to anthrax LF domain I-III peptide panel in immune human donors. Table indicates positive T cell IFNγ ELIspot responses that were seen in 3 or more donors from the human donor cohort described in the Methods, comprising a total of 9 donors in the cutaneous anthrax (Kayseri) group and 10 donors in the AVP vaccinees (UK) group.

In the AVP vaccinated individuals the immunodominant response encompassed five epitopes. Of these peptides, LF_41–60_, LF_417–436_, and LF_437–456_ did not induce a response in any of the HLA transgenics (LF_337–356_ was identified as a cryptic epitope which was identified in the HLA-DQ8 transgenics, data not shown). The T cell responses to the domain I peptide LF_101–120_ was confirmed as an HLA-DQ8 specific response in the transgenic mice.

In the naturally infected donors from Kayseri, however, the T cell response was focused on two LF peptides. In parallel with the epitope hierarchy identified in the AVP vaccinees, a peptide epitope, LF_281–300_, was also identified which did not induce a response in any of the HLA transgenics. The remaining domain II peptide, LF_467–487_, had previously been identified as an immunodominant HLA-promiscuous epitope, capable of eliciting a T cell response from all the HLA transgenics.

We have previously documented immune responses to domain IV in humans [32], however it is interesting to note that, of the epitopes identified in that previous study, in AVP vaccinees, LF_674–693_ has been confirmed as an immunodominant epitope in both HLA-DR1 and HLA-DR15 transgenics, and the peptides LF_574–593_, LF_654–673_ and LF_694–713_ were all identified as immunodominant epitopes in this study, which each elicited a T cell response in a single HLA transgenic strain. Furthermore, the domain IV epitopes previously reported in Turkish naturally infected anthrax patients, LF_694–713_ and LF_714–733_ have both been identified as immunodominant epitopes in HLA-DR15 transgenics. Although the domain IV peptide LF_584–603_ which was a feature of the AVP vaccinee's immune response, did not induce any response in any of the HLA transgenics in this study.

There was very little overlap in responses of the infected and vaccinated human cohorts: the immunodominant, strongly binding epitope, LF_467–486_, was recognized by a high proportion of naturally infected donors, but not vaccinated individuals. This suggests that the peptide is processed and strongly immunogenic during infection, but is not recognized in the response to the protein antigen during immunization. Could epitope differences between the cohorts be explained by the fact that the individuals come from different geographical regions and express different HLA class alleles? We report the HLA-typing of the donors, and indeed the common HLA class II alleles present in the studied region of Turkey are not substantially different from the common alleles in the studied cohort regions of the UK. Ultimately, the number of individuals in this study, powered for functional rather than genetic association studies in its inception and design, is too low to draw conclusions about the possibility that different HLA allele frequencies may drive different preferences for immunodominant epitopes.

**Figure 4:**
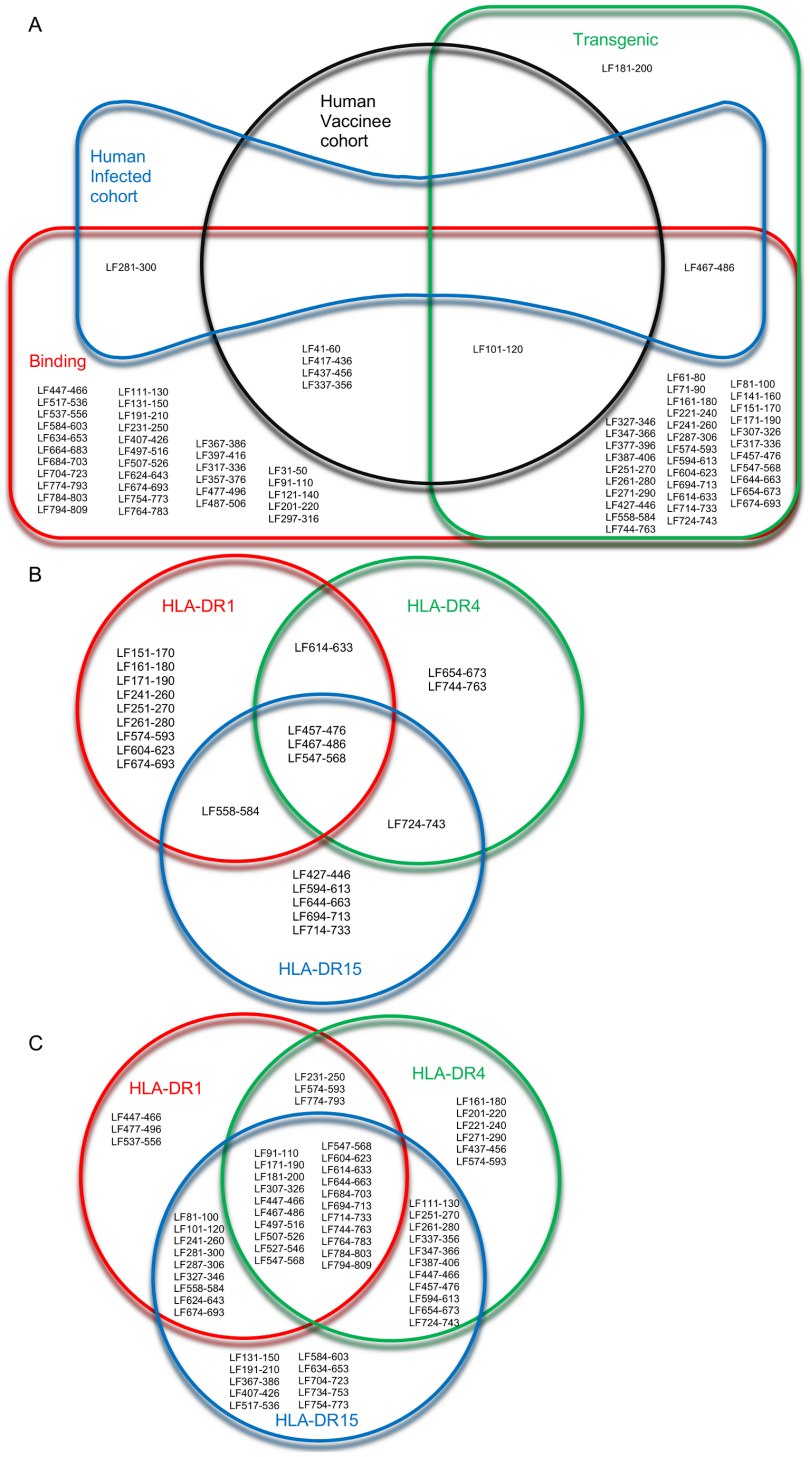
Overview of allele specific and promiscuous epitopes identified by binding affinity, and immunogenicity in HLA transgenic mice and human subjects. The overlapping relationships of the epitopes identified in the HLA transgenic responses, HLA-DR binding affinity studies, and in cohorts of vaccinated and infected humans, were demonstrated in a Euler diagram (A). The HLA-DR restricted epitopes identified in (B) HLA transgenic mice and (C) HLA binding affinity studies were visualised as Venn diagrams, to show allele specific and promiscuous epitopes.

The majority of HLA class II restricted epitopes characterised by this study were identified by more than one experimental system (Figure 4A). The most notable epitope, LF_467–486_ showed strong or moderate HLA-DR binding affinity across a range of alleles, and was immunogenic in HLA-transgenics and infected humans.

A comparison of HLA-DR restricted peptides, showed the overlapping subsets of allele specific and promiscuous epitopes identified by binding affinity (Figure 4C) and immunogenicity in HLA transgenic mice (Figure 4B). Although the binding affinity assays suggest 21 peptides demonstrated strong or moderate promiscuous binding to HLA-DR1, DR4 and DR15 (Figure 4C), only three peptides, LF_457–476_, LF_467–486_ and LF_547–568_ were immunogenic in all three HLA-DR transgenic strains analysed (Figure 4B). It is interesting to note that, according to the binding affinity studies LF_467–486_ and LF_547–568_, but not LF_457–476_, were strong binders to all three HLA-DR alleles (Table 3), demonstrating the importance of validating the immunogenicity of T cell epitopes *in vivo*.

**Table 3:** Summary detailing the immunogenicity of the promiscuous dominant epitopes identified within this work.

LF peptide sequence	HLA-restriction based on CD4 T cell response class II HLA transgenics after priming with LF					Strong relative binding affinity of peptide to HLA-DR molecules							T cell response in infected human cohort	T cell response in vaccinated human cohort
	DR1	DR4	DR15	DQ8	DQ6	DR1	DR3	DR4	DR7	DR11	DR13	DR15		
^457^ HQSIGSTLYNKIYLYENMNI ^476^	+	+	+	+	+	−	+	−	+	−	+	+	−	−
^467^ KIYLYENMNINNLTATLGAD ^486^	+	+	+	+	+	+	+	+	+	+	−	+	+	−
^547^ LENGKLILQRNIGLEIKDVQI ^567^	+	+	+	−	−	+	+	+	+	+	+	+	−	−

It is important to recognise the limitations of this study; the strength of HLA binding is based exclusively on seven HLA-DR alleles, whilst all of the human cohorts presented the peptides through a diverse and heterogeneous mixture of HLA class II alleles. Nonetheless, it is striking that LF_467–486_ not only showed strong binding affinity across all HLA-DR alleles assayed, but all 5 HLA transgenic strains and in infected individuals, showed strong T cell responses to this peptide (Tables 1 and 2), demonstrating a truly promiscuous HLA class II binding and immunogenic nature.

### Immunization of HLA transgenics with an LF fusion protein or a peptide cocktail of LF T cell epitopes confers protection from anthrax challenge

The primary importance of humoral immunity in mediating protection against anthrax has been brought into question by recent studies suggesting that IFNγ producing CD4^+^ T cells play an important role in long lasting immunity [32], [45]. In addition, induction of memory CD4^+^ T cells may feedback not only to cellular immunity, but also aid in the production of toxin neutralising antibodies, Ig class switching and B cell affinity maturation. To determine whether the immunodominant T cell epitopes identified within LF could be incorporated into an epitope string vaccine capable of conferring protection against lethal anthrax challenge in a mouse model, the HLA-DQ8 transgenic mice were immunized with either a fusion protein comprising HLA-DQ8 restricted epitope moieties expressed contiguously after a tetanus toxin helper domain, or a cocktail of the same epitopes as synthetic peptides.

**Figure 5:**
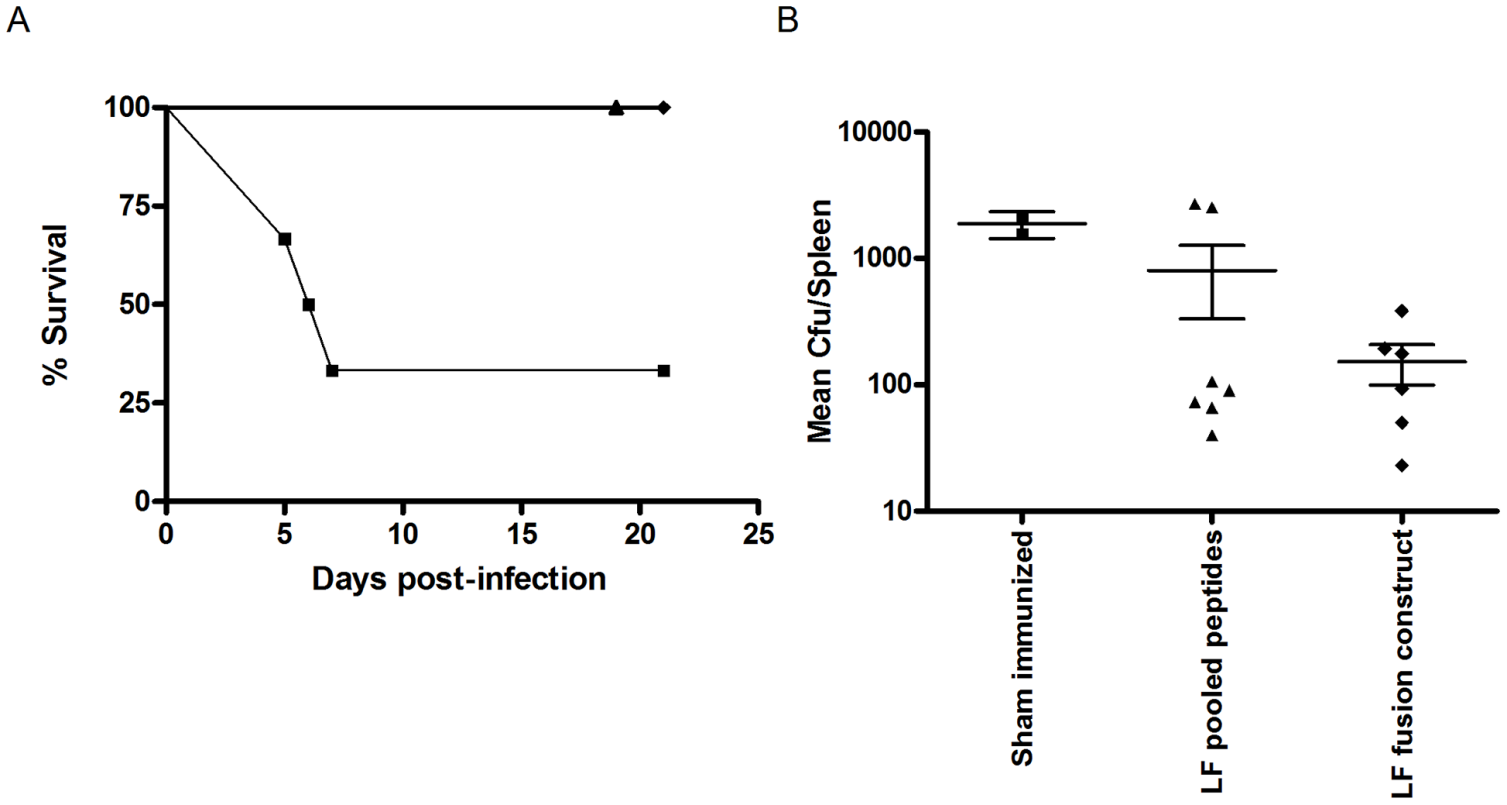
An LF epitope based vaccine which stimulates HLA restricted T cell immune responses may confer protection against *B. anthracis* challenge. Groups of HLA-DQ8 transgenic mice were immunised 3 times, on days 0, 14 and 35 by the intra-peritoneal route, with either an LF fusion construct comprising a tetanus toxin helper domain (aa 865–1120) and 12 confirmed HLA-restricted LF epitopes (n = 6, black diamonds), or a peptide pool of the LF epitopes expressed in the fusion protein (n = 7, black triangles), a control, sham immunised, group was also included in the experiment (n = 6, black squares). All groups were challenged with 10^6^ cfu *B. anthracis* STI by the intra-peritoneal route, on day 77, and monitored daily for survival (A). The impact of infection upon survival was described using Kaplan Meier estimation (A). Spleens were recovered from surviving mice at day 21, (LF fusion protein (n = 6, black diamonds), peptide pool (n = 7, black triangles) and sham immunized mice (n = 2, black squares)), and a mean bacterial count per spleen determined following culture of *B. anthracis* for 24 hours (B). No statistically significant difference was seen between the groups in terms of bacterial burden.

HLA-DQ8 transgenics primed and boosted with 3 doses of an LF fusion construct containing HLA-restricted LF epitopes were fully protected against challenge with 10^6^ cfu *B.anthracis* STI. The naïve, sham immunized group showed a significantly lower survival rate than either the group primed and boosted with 3 doses of the pooled peptides which were expressed in the fusion protein (p>0.01) or the fusion protein (p>0.01) immunized groups (Figure 5A). Only 2/6 naïve mice survived to day 20 post-infection, with a median survival time of 6 days in this group. The bacterial loads recovered from the spleens of surviving mice showed that the immunized mice appeared to clear the infection more successfully than the naïve mice, (naïve group (1883.4+/−317 cfu), peptide cocktail (801.2+/−469 cfu) and LF fusion (153 +/−54 cfu)), however it was not possible to detect a significant difference between groups in terms of bacterial burden (Figure 5B). The high degree of protection against anthrax infection observed in both the immunized groups indicated, not only that the LF fusion protein was capable of conferring the same protective affect as the individual peptides, but also validated the immunoprotective effects of the epitopes identified within this study. Evaluation of peptide-specific responses on a second group of HLA-DQ8 transgenic mice immunized with either LF fusion protein or peptide cocktail showed that the strongest peptide recall in both groups was to LF_467–486_ (data not shown). These data suggest that LF_467–486_ and the promiscous epitopes which were included in these immunisations prime a strong T cell response, playing a role in protection against anthrax.

## Discussion

While considerable attention has been devoted to the profound immune subversion mediated by anthrax toxins [46], recent human studies, including this one, show that anthrax infection can be immunogenic [4]. The role of LT in the disruption of the MAPK signalling pathways, with its consequences for the apoptosis of antigen presenting cells, specifically the lysis of dendritic cells and macrophages, might be expected to subvert host immunity and promote systemic anthrax infection. However, investigation of the inverse relationship between sensitivity to LT and resistance to infection, indicates that mice which possess alleles encoding an LT-sensitive form of Nlrp1b promote a pro-inflammatory response predominantly driven by inflamasome-mediated cell lysis and release of IL-1β [47]–[50]. The associated cell infiltration and cytokine milieu seen in early inflammation may be crucial in driving antigen presentation and T cell priming. Recent studies ranging from asymptomatic seroconversion of wool-workers to our own recent work with near lethal anthrax infection in intravenous drug users, show common themes in terms of strong induction of adaptive immunity [4], [25].

For an infection in which we believe there is a key role of host Th1 immunity, it would be assumed that IgG2a neutralizing antibodies would be an important correlate of protection. However, since the most relevant studies in which this can be analyzed in detail tend to be primate studies based on protection by alum-adjuvanted vaccine, it is the vaccine formulation itself that tends to be the main driver of protective IgG subclasses, both IgG1 and IgG2a being found in the protective response [51].

LF protein boosts PA-specific antibody responses following co-administration [30], [52], and the incorporation of a truncate containing the N-terminal region of LF into a PA plasmid expression vector enhances the PA-specific antibody response [52], while LF truncated proteins are capable of conferring protection against *B. anthracis* aerosol challenge [53], [54]. Thus LF-specific responses may be more important mediators of protective immunity than previously thought. Previous work by our lab has identified LF as a major target of T cell immunity in humans [32], despite the amount of LF released by *B. anthracis* being one-sixth that of PA [55].

Antigen presentation through both HLA-DR and DQ is important in the induction of immunity, and the allelic diversity inherent in these class II molecules shapes the T cell repertoire and influences susceptibility to infection [56]. The reductionist approach of using transgenic models was deployed here as a means of defining HLA restricted T cell responses to immunogenic epitopes of LF. Across the transgenic lines, representing five HLA class II alleles, along with the expected allele specific epitopes, the T cell response showed a number of broad similarities. This was most evident in the response to LF domain II, which produced immunogenic responses in both HLA-DR and DQ transgenic mice following stimulation with either the whole domain, or the individual peptides LF_457–476_ and LF_467–487_, which dominate the T cell response to this domain. These immunodominant epitopes, which were also found to have a high binding affinity for a wide range of HLA-DR molecules, therefore comprise ‘public specificities’ or promiscuous epitopes which are efficiently presented by APCs, to a peptide-MHC specific TCR repertoire, in all HLA transgenics (Table 3).

The C-terminal domain II of LF shows structural homology with the ADP-ribosyltransferase found in the *Bacillus cereus* VIP2 toxin. In conjunction with domains III and IV, domain II forms the active site which is involved in substrate recognition and binding [57]. The amino terminus of the MAPK kinases substrates fit into the LF groove which contains several, conserved, long chain, aliphatic residues [58]. These residues occur in three distinct clusters; the first is composed of Ile_298_, Ile_300_, Ile_485_, Leu_494_, and Leu_514_, the second cluster of residues contains Ile_322_, Ile_343_, Leu_349_, Leu_357_, and Val_362_ which lie at the end of the catalytic groove. The final cluster of aliphatic residues lies close to the domain IV groove; Leu_450_, Ile_467_, Leu_677_, Leu_725_, and Leu_743_ [58]. Both of the immunodominant epitopes LF_457–467_ and LF_467–487_ overlap two of the aliphatic residues, Ile_467_ and Ile_485_ which may have an effect upon the substrate binding of MAPK kinases. It is tantalising to note that the host response focuses on this active site, for which the evolutionary cost of mutation would be high for the pathogen; one must of course note, however, that anthrax is not an obligate human pathogen, is not commonly spread between people and can survive in spore form in soil. Thus, this is not an infection where there is likely to be an overt host-pathogen arms race.

The T cell responses to the peptide LF_547–567_, from domain IV, appeared to be HLA-DR restricted, as only T cells from the DR transgenics, HLA-DR15, HLA-DR4 and HLA-DR1, not the DQ transgenics HLA-DQ6 and HLA-DQ8, responded to this peptide. Domain IV is the catalytically active center of the LF toxin [43], and its protein folds contain a sequence which shares similarity with the zinc-dependant metalloproteases found in the toxin produced by *C. tetani* [59]. Previous work has indicated that this homologous region of the tetanus toxin contains a number of HLA-DR restricted T cell epitopes [60]. The ability of the LF domain IV to readily provoke a recall response in CD4^+^ T cells in the HLA-DR transgenics, suggests that the immune response to this particular domain of the LF protein is also dominated by HLA-DR restricted T cells. It has been observed that mutations in the sequence coding for domain IV disrupts the substrate binding groove created by domains II, III and IV, eliminating the peptidase activity of LF, and thereby abrogating its toxicity [61]. The putative zinc binding site [HEFGHAV] which occurs between the amino acid residues LF_686_ and LF_690_ [62] was only a feature of the HLA-DR1 transgenic response to LF_674–693_

A number of immunodominant epitopes identified within LF showed broad HLA binding characteristics, most notably the domain II epitopes LF_457–476_ and LF_467–487_ which showed strong binding across a range of HLA-DR molecules as well as the preponderance of epitopes from domain IV which were presented by HLA-DR. The strength of HLA binding does not however appear to predict the immunodominance of the peptide epitope. This contrasted with a number of studies, which have described a strong correlation between the affinity of binding and the ability of a peptide to be presented by a particular MHC molecule resulting in an immunodominant T cell response [63]–[66].

Of the five HLA strains challenged with domain I peptides, only the HLA-DR1 and HLA-DQ8 transgenics showed CD4^+^ T cell responses to peptide epitopes from this domain. Domain I binds with high affinity to the proteolytically active 63 kD PA heptamers which are responsible for the membrane-translocation of the anthrax toxins [67]. Over the first 250 residues, this domain shares significant sequence identity and similarity with domain I of EF [43]. The N-terminal sequence of both toxins contain a common domain for PA binding, which in LF has been shown to be sufficient to act alone as a carrier for delivery of heterologous proteins across membranes in the presence of PA [68]. The sequence homology between the two toxins within domain I was demonstrated by the use of LF induced antibodies which were cross-reactive with EF [69]. One of the cross-reactive epitopes LF_265–274_ (which corresponds to EF_257–268_), overlapped with the HLA-DR1 restricted T cell epitopes LF_251–270_ and LF_261–280_, indicating that these epitopes have the potential to induce a neutralising antibody response to both LF and EF as well as the T cell response, making them interesting candidates for inclusion in a polyepitopic anthrax vaccine. In contrast to domain IV peptides, epitopes in this domain are presented in the context of both DR and DQ, although there appears to be minimal overlap in the specific peptides presented.

None of the transgenics showed immunodominant CD4^+^ T lymphocyte responses against the individual peptides which make up domain III. The helix bundle which makes up domain III is inserted into domain II, and may have arisen from repeated duplications of a structural element of domain II [40]. Although these domains share elements of their structure and function, the CD4^+^ T cell response to each is very different. Domain III appears to be a hidden or infrequent target of the immune response.

Most vaccine strategies against anthrax have concentrated on PA, although the UK AVP vaccine, which contains both PA, and lower levels of LF, stimulates LF specific antibodies [70]–[72], while exposure to natural infection results in a faster, antibody response to LF than PA [73]. It was discovered that the magnitude of the CD4^+^ T cell response to LF antigens was greater in naturally infected individuals than in vaccinees [32]. The T cell immunity to LF, particularly domain IV, identified in naturally infected individuals is in contrast to the expected response to LF exposure, especially in the context of infection, which might be expected to impair the T cell memory of *B. anthracis* in survivors of natural infection. Taking into account all the HLA-DP, DQ and DR products, as well as inter and intra isotypic mixed pairs, a heterozygous human can present peptides for CD4^+^ T cell recognition on up to 12 different class II molecules. It is therefore interesting to note that despite the immunogenetic heterogeneity seen in human populations, which along with differences in exposure to the antigen, might be expected to complicate the pattern of epitopes recognised by the human cohorts studied, amongst the naturally infected individuals, the immunodominant promiscuous LF_467–487_ epitope was one of the main targets of a strong CD4^+^ T cell response.

Some CD4 epitopes identified in human vaccinees were not seen in the naturally infected individuals; it might be expected that some epitopes present in the context of vaccination would be lost on infection. It is unclear whether such changes in antigen focus reflect differential antigen processing of pathogen proteins encountered in vaccination in contrast to infection, or if this represents an artefact of the repeated AVP vaccinations which may skew the cytokine environment during induction of the immune response, impacting upon the T cell epitope repertoire [74]. Humans exposed to LF following cutaneous anthrax infection generate robust long-term T cell memory to *B. anthracis* epitopes, in many cases several years after the initial infection event. The T cell response in these naturally infected individuals showed significantly elevated levels of the pro-inflammatory cytokines associated with Th1, Th2, Th9 and Th17 subsets compared to vaccinees and naïve controls [32]. The inhibitory effects of both LT and ET upon expression of the activation markers CD25 and CD69 and the secretion of the pro-inflammatory cytokines IL-2, IL-5, TNFα, and IFNγ by human T cells has been described *in vitro* [75], [76]. Murine lymphocytes show impaired TCR-mediated activation and T cell dependent production of IL-3, IL-4, IL-5, IL-6, IL-10, IL-17, TNFα, IFNγ and GM-CSF following exposure to LT and ET [77]. However, the cellular immunity we have identified within the naturally infected humans indicates that, although *in vitro* exposure to ET has been implicated in immune deviation towards both the Th2 and Th17 pathways [78], [79], the human immune response against LF encompasses a strong IFNy response. It was suggested to the authors that, since the predominant mechanism of protective immunity to anthrax toxin is antibody neutralization, it is possible that T follicular helper cells, characterised by the co-production of IFNγ and IL-21 and vital for B cell help, may be important here. In response to the reviewer's suggestion, we have considered the notion that this is a TFH response by looking for IL-21 accompanying the IFNγ response in each of our donor responses, but, as we now report, detected none; we therefore consider it less likely that these are predominantly TFH cells.

Despite the presence of many potential peptide epitopes within LF, the elicited T cell response indicates that immunodominant LF epitopes are concentrated in domains II and IV. The immunodominant epitopes identified within these domains appear to comprise essential residues of LF which are critical for efficient catalytic activities and the execution of substrate cleavage. We therefore suggest that a number of the immunodominant epitopes which we have identified represent regions of the LF protein in which the cost of mutation to *B. anthracis* would be too high, due to the resultant loss of function. The identification of the immunodominant epitope LF_467–487_, which represents a rare truly promiscuous antigen, capable of binding strongly to multiple diverse HLA alleles, and which is also a feature of a robust T cell response in naturally infected individuals, presented us with a unique opportunity to develop a polyepitopic vaccine in which each epitope is promiscuous, or covers a number of HLA alleles. This increases the chance that each individual in a genetically heterogeneous population acquires immunity to multiple epitopes from a pathogen, thus offering increased protection to a population. We found that the 12 HLA-restricted LF epitopes, either incorporated as a fusion construct or as a peptide pool, conferred protection against lethal challenge with *B. anthracis*. In addition to their defined role in the T cell response to LF antigens *in vitro*, this suggests that the epitopes we have described here are capable of priming a strong, long-lasting T cell response that play a role in protection against anthrax. Further work to attribute this protection to a specific response, through both cellular and humoral markers, would be of merit in determining the potential of the LF fusion protein as a future anthrax vaccine candidate.

The nature of anthrax infection and the need to evolve tractable strategies, notably in a biodefense setting, has necessarily led to a reliance on a program of PA vaccines tested in primate challenge studies. Study of immunity in naturally-exposed humans, who seem to be immune to reinfection, raises the possibility of learning from these immune repertoires, including the role of LF as a target.

## Materials and Methods

### Expression and purification of LF antigens

Recombinant full-length LF (rLF) and individual domains were produced in an *E. coli* expression system as previously described [80]. In brief, the cysteine residue at position 687 was replaced with glutamic acid to produce a biologically inactive form of LF. The gene sequence of LF was codon optimized for expression in *E. coli* (GenScript, USA) to allow for the high AT nucleotide content of the protein. Using the pQE30 expression system (Qiagen, Germany) the full length LF and LF domain sequences were cloned and expressed from *E. coli* as recombinant N-terminal histidine-tagged proteins. Bacterial pellets were disrupted using a French press, and the target proteins recovered by centrifuging for 20 minutes at 45000×*g* at 4°C. These were then incubated with Talon metal affinity resin (Clontech, USA) to bind the N-terminal histidine tag. The proteins were eluted from this resin at 4°C by washing with protein elution buffer. Protein concentration was determined using a bicinchoninic acid (BCA) protein assay protocol (Pierce, Thermo Scientific, USA) and dialyzed against HEPES buffer, using a 10000 molecular weight cut-off dialysis cassette (Pierce, Thermo Fisher Scientific, USA), to a final endotoxin level of <4 EU/mg. A synthetic peptide panel, HPLC purified to a purity of ≥98% purity, comprising of 20mer amino acids overlapping by 10 amino acids encompassing the full-length sequence of LF were obtained from a commercial supplier (Abgent, USA). All peptides were resuspended in DMSO at 25 mg/ml.

### HLA transgenic mice

HLA class II transgenic mice carrying genomic constructs for HLA-DRA1*0101/HLA-DRB1*0101 (HLA-DR1), HLA-DRA1*0101/HLA-DRB1*0401 (HLA-DR4), HLA-DRA1*0101/HLA-DRB1*1501 (HLA-DR15), HLA-DQA1*0301-DQB1*0302 (HLA-DQ8) and HLA-DQA1*0102/HLA-DQB1*0602 (HLA-DQ6), crossed for more than six generations to C57BL/6 H2-Aβ^00^ mice, were generated and described previously [81]–[86]. All experiments were performed in accordance with the Animals (Scientific Procedures) Act 1986 and were approved by local ethical review.

### Ethics statement

All mouse experiments were performed under the control of UK Home Office legislation in accordance with the terms of the Project License granted for this work under the Animals (Scientific Procedures) Act 1986 having also received formal approval of the document through the Imperial College Ethical Review Process (ERP) Committee. Human blood samples for the Kayseri (Turkey) component of this study were obtained with full review and approval by The Ethics Committee of the Faculty of Medicine, Erciyes University; all participants were adults over 18 year old. Participants were given a full, verbal explanation of the project and written consent was obtained from all those who elected to participate. Human vaccinees based at DSTL, Porton Down, participated in the context of a study protocol approved by the CBD IEC (Chemical and Biological Defence Independent Ethics Committee); the subjects were all adults aged over 18 years and all provided written, informed consent. Healthy control blood samples were collected under the approval of Ethics REC reference number 08/H0707/173.

### Live *B. anthracis* challenge

HLA-DQ8 transgenic mice were challenged intra-peritoneally with 10^6^ colony forming units of *B. anthracis* STI strain. The animals were monitored daily for 20 days post-infection, and post-mortem spleens were homogenized in 1 ml of PBS prior to plating out at a range of dilutions onto L-agar plates. Colonies were counted after 24 hours culture at 37°C, and the mean bacterial count per spleen was determined.

### Patient samples

Leukocytes were isolated from human peripheral blood samples and stimulated as described previously [32]. In brief, sodium heparinised blood was collected with full informed consent from 9 Turkish patients treated for cutaneous anthrax infection within the last 8 years. (Ericyes University Ethical Committee), 10 volunteers routinely vaccinated every 12 months for a minimum of 5 years with the UK Anthrax Vaccine Precipitated (AVP) vaccine (UK Department of Health under approval by the Convention on Biological Diversity Independent Ethics Committee for the UK Ministry of Defence), and 10 age-matched healthy controls with no known exposure to anthrax antigens (Ethics REC reference number 08/H0707/173). PBMCs were prepared from the blood using Accuspin tubes (Sigma, Dorset, UK) and washed twice in AIM-V serum free medium (Life Technologies, UK). Cells were counted for viability and resuspended at 2×10^6^ cells/ml.

### LF epitope mapping and confirmation

Mice were immunized in the hind footpad with 50 µl of 12.5 µg rLF, LF peptides, individual LF domains or a control of PBS, emulsified in an equal volume of Titermax Gold adjuvant (Sigma-Aldrich, USA). After 10 days, immunized local draining popliteal lymph nodes were removed and disaggregated into single cell suspensions. Lymph node cells (3.5×10^6^/ml) were challenged with 25 µg/ml of either recombinant full-length LF, the 4 domains which comprise the LF protein, or the overlapping 20mer peptides covering the full-length LF sequence. This generated a map of the entire LF protein sequence. To confirm the immunodominant epitopes identified by this large scale mapping, mice were then immunized subcutaneously with 12.5 µg of the individual LF peptides in Titremax adjuvant. After 10 days the lymph node cells were challenged *in vitro* with 25 µg/ml of the recombinant full-length LF and the immunising and two flanking LF peptides.

In the human T cell assays, the peptide library was prepared in a matrix comprising 6 peptides per pool, so that each peptide occurred in 2 pools but no peptides occurred in the same two pools. This allowed the determination of responses to individual peptides. The in-well concentration of each peptide was 25 µg/ml and total peptide concentration per well was 150 µg/ml.

### Lymphocyte proliferation assay

Leukocytes were resuspended at 3.5×10^6^ cells/ml in HL-1 media (1% L-Glutamine, 1% Penicillin Streptomycin, 2.5% β-Mercaptoethanol) and 100 µl/well was plated out in triplicate on 96 well Costar tissue culture plates (Corning Incorporated, USA). The cells were stimulated with 100 µl/well of, appropriate antigen, positive controls of 5 µg/ml Con A (Sigma-Aldrich, USA) or 25 ng/ml of SEB (Sigma-Aldrich, USA) or negative controls of medium alone. The plates were incubated at 37°C, 5% CO_2_ for 5 days. Eight hours prior to harvesting, 1 µCi/well of [^3^H]-Thymidine (GE Healthcare, UK) was added. The cells were harvested onto fiberglass filtermats (PerkinElmer, USA) using a Harvester 96 plate harvester (Tomtec, USA) and counted on a Wallac Betaplate scintillation counter (EG&G Instruments, Netherlands). Results were expressed as stimulation index (SI) (cpm of stimulated cells divided by cpm of negative control cells). An SI of ≥2.5 was considered to indicate a positive proliferation response.

### IFNγ ELISpot assay

Quantification of murine antigen-specific IFNγ levels was carried out by ELISpot (Diaclone) analysis of T cell populations directly *ex vivo*. Hydrophobic polyvinyldene difluoride membrane-bottomed 96-well plates (MAIP S 45; Millipore) were pre-wetted with 70% ethanol, washed twice and then coated with anti-IFNγ monoclonal antibody at 4°C overnight. After blocking with 2% skimmed milk, plates were washed and 100 µl/well of antigen was added in triplicates. For each assay a medium only negative and a positive control of SEB (25 ng/ml) were included. Wells were seeded with 100 µl of 2×10^6^cells/ml in HL-1 medium (1% L-Glutamine, 1% Penicillin Streptomycin, 2.5% β-Mercaptoethanol) and plates were incubated for 72 h at 37°C with 5% CO_2_. Plates were washed twice with PBS Tween 20 (0.1%) then incubated with biotinylated anti-INFγ monoclonal antibody. Plates were washed twice with PBS Tween 20 (0.1%), and then incubated with streptavidin-alkaline phosphatise conjugate, washed and then treated with 5-bromo-4-chloro-3-indolyl phosphate and nitroblue tetrazolium (BCIP/NBT) and spot formation monitored visually. The plate contents were then discarded and plates were washed with water, then air-dried and incubated overnight at 4°C to enhance spot clarity. Spots were counted using an automated ELISpot reader (AID), and results were expressed as delta spot forming cells per 10^6^ cells (ΔSFC/10^6^) (SFC/10^6^ of stimulated cells minus SFC/10^6^ of negative control cells). The results were considered positive if the ΔSFC/10^6^ was more than two standard deviations above the negative control.

Human T cell INFγ levels were quantified by ELISpot (Diaclone) as previously described [32]. In brief, the plates were prepared in a similar manner to the murine ELISpots and following addition of antigen to the wells (with each peptide represented in two separate triplicates) they were frozen at −80°C until use. Wells were seeded with 100 µl of human PBMCs at 2×10^6^ cells/ml (range; 1.6×10^6^–2.1×10^6^ cells/well) in AIM-V medium and plates were incubated for 72 hours at 37°C with 5% CO_2_. 50 µl supernatant was removed from each well for further determination of cytokines, the remaining plate contents were then discarded and plates were washed with PBS Tween 20 (0.1%) and incubated with biotinylated anti–INFγ, followed by a further wash and the addition of streptavidin-alkaline-phosphatase conjugate. Following a final wash, plates were developed by addition of BCIP/NBT. Spots were counted using an automated ELISpot reader (AID), and results were expressed as delta spot forming cells per 10^6^ cells (ΔSFC/10^6^) (SFC/10^6^ of stimulated cells minus SFC/10^6^ of negative control cells). The results were considered positive if the ΔSFC/10^6^ was more than two standard deviations above the negative control and ≥50 spots. IL-21 release from peptide-stimulated donor T cell cultures was determined by ELISA (eBiosciences).

### HLA peptide binding assay

Competitive ELISAs were used to determine the relative binding affinity of LF peptides to HLA-DR molecules, as previously described [87]. Briefly, the HLA-DR molecules were immunopurified from homozygous EBV-transformed lymphoblastoid B cell lines by affinity chromatography. The HLA-DR molecules were diluted in HLA binding buffer and incubated for 24 to 72 hours with an appropriate biotinylated reporter peptide, and a serial dilution of the competitor LF peptides. A control of unlabeled reporter peptides was used as a reference peptide to assess the validity of each experiment. 50 µl of HLA binding neutralisation buffer was added to each well and the resulting supernatants were incubated for 2 hours at room temperature in ELISA plates (Nunc, Denmark) previously coated with 10 µg/ml of the monoclonal antibody L243. Bound biotinylated peptide was detected by addition of streptavidin-alkaline phosphatase conjugate (GE Healthcare, Saclay, France) and 4-methylumbelliferyl phosphate substrate (Sigma-Aldrich, France). Emitted fluorescence was measured at 450 nm post-excitation at 365 nM on a Gemini Spectramax Fluorimeter (Molecular Devices, St. Gregoire, France). LF peptide concentration that prevented binding of 50% of the labeled peptide (IC_50_) was evaluated, and data expressed as relative binding affinity (ratio of IC_50_ of the LF competitor peptide to the IC_50_ of the reference peptide which binds strongly to the HLA-DR molecule). Sequences of the reference peptide and their IC50 values were as follows: HA 306–318 (PKYVKQNTLKLAT) for DRB1*0101 (4 nM), DRB1*0401 (8 nM), and DRB1*1101 (7 nM), YKL (AAYAAAKAAALAA) for DRB1*0701 (3 nM), A3 152–166 (EAEQLRAYLDGTGVE) for DRB1*1501 (48 nM), MT 2–16 (AKTIAYDEEARRGLE) for DRB1*0301 (100 nM) and B1 21–36 (TERVRLVTRHIYNREE) for DRB1*1301 (37 nM). Strong binding affinity was defined in this study as a relative activity <10, and a moderate binding affinity was defined as a relative activity <100.

### Generation and evaluation of an LF epitope fusion protein

A fusion protein comprising HLA-restricted T cell epitopes from LF downstream of the universal T cell helper domain from the tetanus toxin C fragment (aa 865–1120) was designed and codon optimized to reflect *Salmonella enterica Typhi* codon usage (GenScript Corp). This construct was expressed as a recombinant N terminal histidine tagged protein on the commercially available expression system pQE30 in *E. coli* M15 (Qiagen). The LF epitopes included in the fusion protein were: LF_101–120_, LF_151–170_, LF_261–280_, LF_467–486_, LF_547–567_, LF_574–593_, LF_614–633_, LF_654–673_, LF_674–693_, LF_714–733_, LF_724–743_ and LF_744–763_. Briefly, cultures derived from a single colony were grown overnight at 37°C in LB broth with antibiotic selection. Overnight cultures were subcultured in fresh LB broth until they reached an OD_600_ of 0.550–0.600. To induce protein expression, isopropyl β-D-thiogalactopyranoside (IPTG) was added to a final concentration of 1 mM. Cultures were then incubated at 25°C (200 rpm) for 16 hours. Cells were harvested by centrifugation at 10,000 *g* at 4°C for 20 minutes. His-tagged fusion proteins were purified from bacterial pellets under denaturing conditions; all steps were conducted at 4°C unless otherwise stated. The bacterial pellet was resuspended in suspension buffer (SB) (50 mM NaH_2_PO_4_, 300 mM NaCl, pH 7) by gentle pipetting until a homogenous suspension was obtained. Phenylmethanesulfonylfluoride (PMSF) and lysozyme (Sigma- Aldrich, St. Louis, MO) were added to final concentrations of 1 mM and 0.25 mg/mL respectively. The suspension was stirred for 20 minutes before the addition of deoxycholic acid (Sigma- Aldrich, St. Louis, MO) to a final concentration of 1 mg/mL. The lysate was incubated at 37°C, with occasional stirring, until viscous, and DNase I added to a concentration of 0.01 mg/mL. The lysate was stored at room temperature until no longer viscous before centrifugation at 10,000 *g* for 20 minutes. The resulting pellet was washed three times in SB containing 1% Triton X-100, then washed in SB containing 2M urea before resuspension in SB containing 8M urea and centrifugation at 13,000 *g* for 15 minutes. The supernatant was collected and incubated with Talon metal affinity resin (Clontech Laboratories) to bind the N terminal histidine tag. Following washing of the resin with SB containing 6 M urea, the protein was recovered at 4°C in elution buffer (150 mM imidazole, 50 mM sodium phosphate and 300 mM NaCl, 6 M urea, pH 7). Eluate was dialyzed using a 10,000 MW cut off dialysis cassette (Pierce, Thermo Scientific) in dialysis buffer (DB) (10 mM HEPES, 50 mM NaCl, 400 mM L-Arginine, pH 7.5) containing sequentially decreasing concentrations of urea for 1 hour periods. Finally, eluate was dialyzed against 4 L HEPES buffer (10 mM HEPES, 50 mM NaCl, pH 7.5). Protein identity was confirmed by SDS-PAGE and Western Blot analysis (Bio-Rad Laboratories). Protein bands were detected by staining with Coomassie Blue after electrophoretic transfer onto polyvinylidene difluoride membranes (Millipore) by Ni-NTA HRP Conjugate (QIAgen Inc.). The protein was of expected size and was recognized by specific antibodies. The endotoxin content of the different protein preparations was determined by the Limulus amoebocyte lysate linetic-QCL assay according to the manufacturer's instructions (Lonza). Protein concentrations were determined using a BCA protocol (Pierce, Thermo Scientific) [88].

The complete amino acid sequence of the fusion protein is: MKNLDCWVDNEEDIDVILKKSTILNLDINNDIISDISGFNSSVITYPDAQLVPGINGKAIHLVNNESSEVIVHKAMDIEYNDMFNNFTVSFWLRVPKVSASHLEQYGTNEYSIISSMKKHSLSIGSGWSVSLKGNNLIWTLKDSAGEVRQITFRDLPDKFNAYLANKWVFITITNDRLSSANLYINGVLMGSAEITGLGAIREDNNITLKLDRCNNNNQYVSIDKFRIFCKALNPKEIEKLYTSYLSITFLRDFWGSDVLEMetYKAIGGKIYIVDGDYVYAKEGYEPVLVIQSSEDYQHRDVLQLYAPEAFNYMetDKFKIYLYENMetNINNLTATLGADLENGKLILQRNIGLEIKDVQIEYIRIDAKVVPKSKIDTKIQKLITFNVHNRYASNIVESAYYLVDGNGRFVFTDITLPNIAEQYTHQDEIYEQVHSKGLYVAVDDYAGYLLDKNQSDLVTNSKKFIDIFKEEGSNLTSYGRSEGFIHEFGHAVDDYAGYLL.  Mice transgenic for HLA-DQ8 were immunized with 25 µg of fusion protein, or alternatively with a peptide pool consisting of 25 µg of each peptide represented in the fusion protein (total concentration 300 µg peptide), control mice were sham-immunized with PBS. All immunizations were adjuvanted 1∶1 in Titremax Gold and administered by the i.p. route (0.1 mL). Mice were immunized on days 0, 14 and 35 prior to challenge with *B. anthracis* STI strain on day 77.

## Supporting Information

Figure S1 **HLA-DR4 transgenic mice sham immunized with a PBS control do not generate a response to either the LF protein, its domains, or the individual peptides which make up the entire protein.** HLA-DR4 transgenic mice (n = 3) were immunised in the footpad with PBS adjuvanted with Titermax Gold. Popliteal lymph nodes were harvested on day 10 and stimulated with 25 µg of either (A) the whole LF protein or the individual domains or (B) the 10mer peptides in the LF peptide library (LF_31–809_). IFNγ production was assayed by ELISpot development and enumeration after 3 days stimulation. Data is represented as scatter plots, showing the responses of mice as the stimulation index (SI) calculated as the mean IFNγ production of triplicate wells in the presence of peptide divided by the mean IFNγ production in the absence of antigen. Responses were considered positive if the response was ≥2 SD above the cells plus medium control.
(TIF)
