# Supplementary figures and images for: Anthrax Lethal Factor as an Immune Target in Humans and Transgenic Mice and the Impact of HLA Polymorphism on CD4+ T Cell Immunity
Source: PLoS Pathog. 2014 May 1;10(5):e1004085. doi: 10.1371/journal.ppat.1004085 (PMC4006929; doi:10.1371/journal.ppat.1004085)

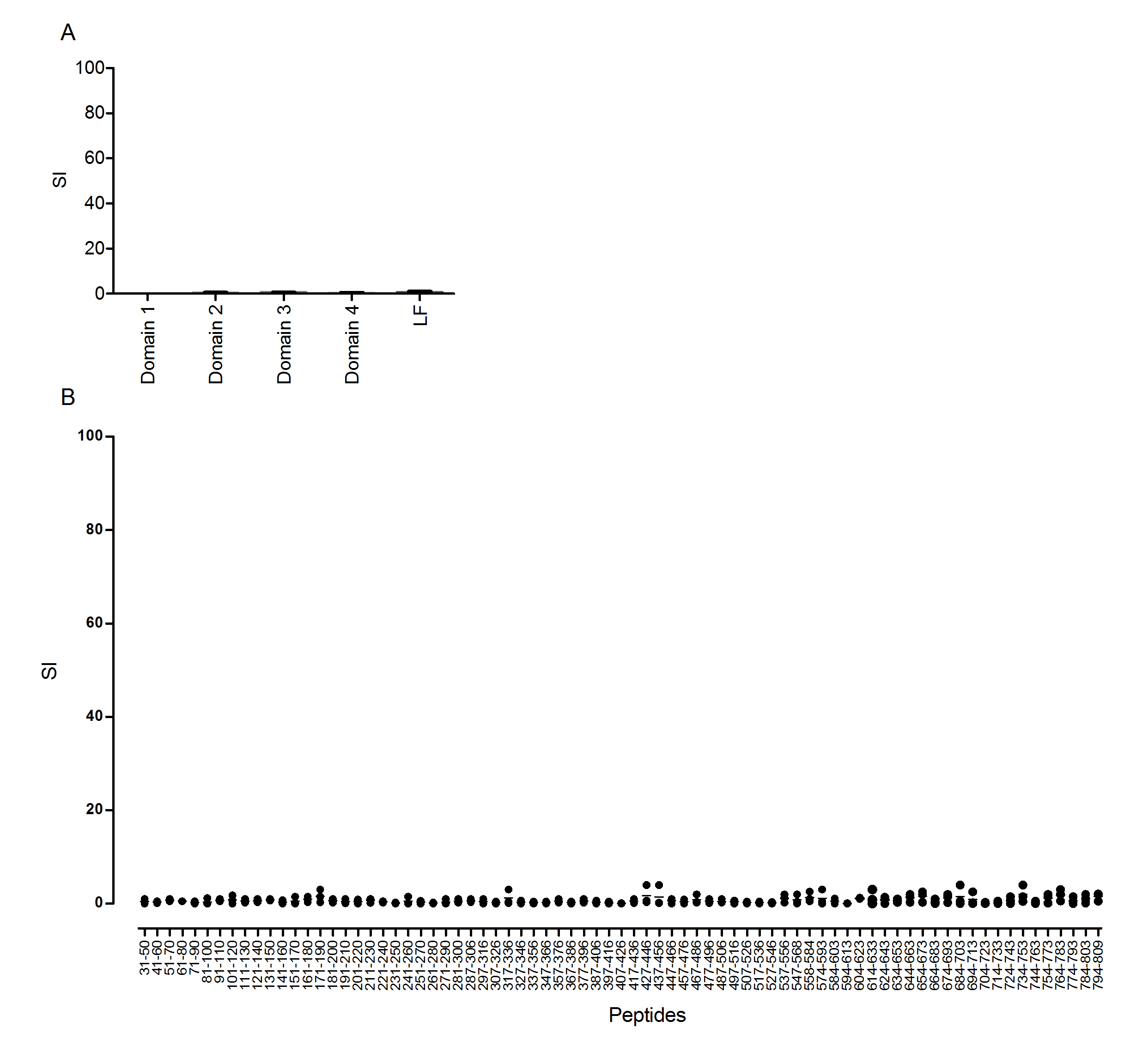

Supplement: Figure S1 — HLA-DR4 transgenic mice sham immunized with a PBS control do not generate a response to either the LF protein, its domains, or the individual peptides which make up the entire protein. HLA-DR4 transgenic mice (n = 3) were immunised in the footpad with PBS adjuvanted with Titermax Gold. Popliteal lymph nodes were harvested on day 10 and stimulated with 25 µg of either (A) the whole LF protein or the individual domains or (B) the 10mer peptides in the LF peptide library (LF31–809). IFNγ production was assayed by ELISpot development and enumeration after 3 days stimulation. Data is represented as scatter plots, showing the responses of mice as the stimulation index (SI) calculated as the mean IFNγ production of triplicate wells in the presence of peptide divided by the mean IFNγ production in the absence of antigen. Responses were considered positive if the response was ≥2 SD above the cells plus medium control. (TIF) [file ppat.1004085.s001.tif]
